# Design of a platform technology for systemic delivery of siRNA to tumours using rolling circle transcription

**DOI:** 10.1038/ncomms8930

**Published:** 2015-08-06

**Authors:** Mihue Jang, Jong Hwan Kim, Hae Yun Nam, Ick Chan Kwon, Hyung Jun Ahn

**Affiliations:** 1 Center for Theragnosis, Biomedical Research Institute, Korea Institute of Science and Technology, Seongbuk-Gu, Seoul 136-791, South Korea; 2 Department of Biochemistry and Molecular Biology, University of Ulsan College of Medicine, 388-1 Pungnap-2 dong, Songpa-Gu, Seoul 136-736, South Korea

## Abstract

For therapeutic applications of siRNA, there are technical challenges with respect to targeted and systemic delivery. We here report a new siRNA carrier, RNAtr NPs, in a way that multiple tandem copies of RNA hairpins as a result of rolling circle transcription (RCT) can be readily adapted in tumour-targeted and systemic siRNA delivery. RNAtr NPs provide a means of condensing large amounts of multimeric RNA transcripts into the compact nanoparticles, especially without the aid of polycationic agents, and thus reduce the risk of immunogenicity and cytotoxicity by avoiding the use of synthetic polycationic reagents. This strategy allows the design of a platform technology for systemic delivery of siRNA to tumour sites, because RCT reaction, which enzymatically generates RNA polymers in multiple copy numbers at low cost, can lead to directly accessible routes to targeted and systemic delivery. Therefore, RNAtr NPs suggest great potentials as the siRNA therapeutics for cancer treatment.

## Abstract (web summary)

RNA interference has provided a promising tool to suppress the expression of specific genes associated with human diseases. Here, the authors present a platform technology for the systemic delivery of siRNA to tumour sites using rolling circle transcription.

## Graphical abstract

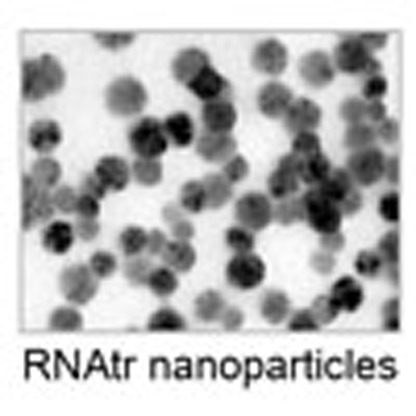

RNA interference (RNAi) is an endogenous regulatory pathway that is triggered by short interfering RNA (siRNA) or microRNA, and by the activated RNAi, siRNA can silence the expression of any genes with high efficiency^1^. Particularly, RNAi has provided a promising tool to suppress expression of specific genes associated with human diseases such as cancers^2^,^3^, viral infection^4^,^5^ and genetic disorders^6^. The approach that utilizes siRNA as the potential drugs has attracted great attention, but there are still critical issues to be addressed before its therapeutic applications. For example, the naked siRNA is rapidly cleared during body circulation due to its low molecular weight and enzymatic degradation^7^. Also, siRNA has no functional moiety targeted to the sites of interest and its negative charge leads to poor cellular uptake owing to the electrostatic repulsion between siRNA and cell membrane^7^. Therefore, an efficient delivery carrier for exogenous siRNA is a prerequisite of its clinical applications.

As an effort to efficiently deliver siRNA, it has been widely explored to complex or encapsulate siRNA with carriers such as polymers^8^,^9^, liposomes^5^,^10^ and dendrimers^11^. However, these conventional methods have critical issues to inevitably introduce a significant amount of synthetic polycationic reagents that may cause undesirable immunogenicity, cytotoxicity or nonspecific accumulation in the uncontrolled manner^12^,^13^, although there have been attempts to modify the synthetic carriers for keeping a balance between their complexing properties and toxicities^14^. Considering the amounts of siRNA loaded into their carriers, low negative charge and intrinsic stiffness of double-stranded siRNA are another barrier to facilitate high cargo capacity^15^. Recently, efficient delivery methods for high cargo capacity have been devised by hybridization of sticky siRNA^16^, polymerization of siRNA via chemical linkages^17^,^18^ and enzymatic RNA polymerization^19^, but even these methods cannot avoid incorporating the synthetic polycationic reagents such as polyethylenimine (PEI). In addition to these issues, it is significantly important to achieve the systemic and targeted delivery of siRNA to specific cells/tissues for therapeutic applications, while minimizing potentially harmful side effects^20^. Recent progresses in the delivery systems involve conjugating siRNA with selected molecules (for example, cholesterol^21^, *N*-acetylgalactosamine^22^ and antibody^23^) through chemical cross-linking; encapsulating siRNA in lipid nanoparticles^24^; developing dynamic polyconjugates for improving the release of siRNA from endosomes^25^; and chemically linking or co-transcribing siRNA with aptamers^26^. The selection of siRNA carrier systems depends on the type of target cells and the properties of siRNA as well as the delivery routes for *in vivo* application.

One of RNA synthetic methods, enzymatic RNA polymerization via rolling circle transcription (RCT), has reported the effective RNA synthetic method for multimeric siRNA, and, particularly, when rationally designed, the polymerized RNA self-assembles into microsponges 2 μm in diameter^19^. Although the RNA microsponge-like particles hardly showed cellular uptake in the cancer cell line without the aid of polycationic reagents PEI, due to their large size and high negative charge, the enzymatic RCT reaction has great merits to generate RNA polymers in multiple copy numbers at low cost when compared with chemical synthesis of siRNA.

To overcome the critical problems caused by synthetic polycationic reagents in the conventional delivery systems, we here report the synthesis of RNA/DNA hybrids that self-assemble into nanoparticles without the aid of polycationic agents; the polymerized RNAs that contain multiple tandem copies of hairpins via RCT are sequentially hybridized with DNA–cholesterol (DNA–Chol) and folate–DNA (FA–DNA) conjugates through Watson–Crick base pairing, yielding the self-assembled RNA transcripts/DNA–Chol/FA–DNA nanoparticles (RNA transcripts nanoparticles, RNAtr NPs). As expected, RNAtr NPs play dual roles as RNAi drug and carrier in that RNAtr NPs protect siRNA against nucleases attack and polyanions interference during transport to the cytoplasm, while generating siRNA with cytoplasmic Dicer enzymes after transfection. RNAtr NPs show a folate receptor-dependent cellular uptake in the *in vitro* studies, and consistently accumulate at tumour sites after systemic administration, resulting in robust gene silencing. In this study, we demonstrate that RCT reaction can be efficiently adopted for systemic delivery of siRNA to tumour sites, and suggest that our design of RNAtr NPs has great potentials as a platform technology for the siRNA therapeutics including anticancer drugs.

## Results

### Synthesis of self-assembled RNA/DNA hybrids

**Figure 1:**
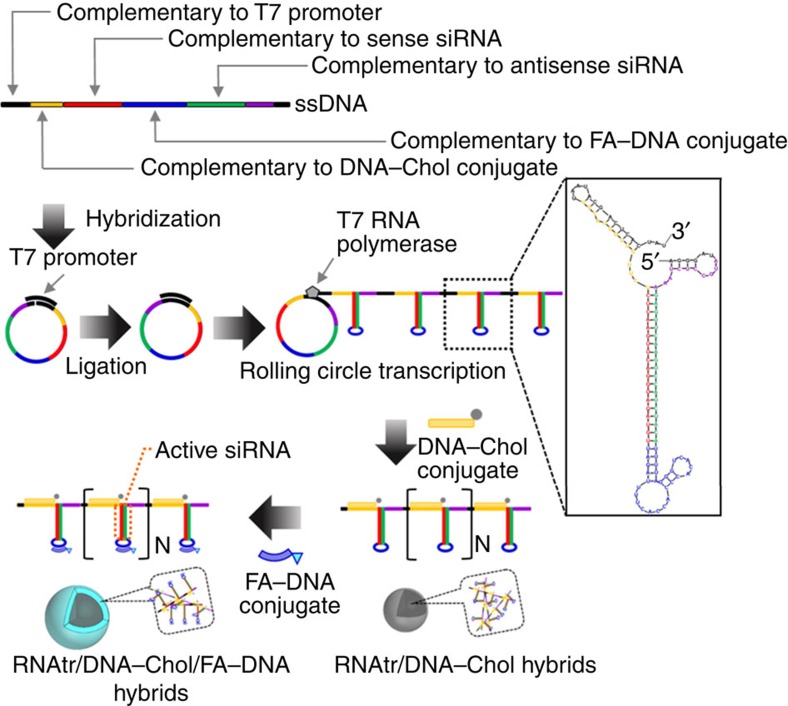
A schematic diagram demonstrating self-assembly of RNAtr nanoparticles. RNA transcripts enzymatically polymerized by rolling circle transcription are composed of multiple tandem copies of RNA hairpins containing sense and antisense sequences of target siRNA duplex. Through RNA/DNA base pairing, DNA–Chol conjugates are hybridized with the RNA transcripts, forming the amphiphilic RNAtr/DNA–Chol hybrids that can self-assemble into the highly condensed nanoparticles. Sequential hybridization of FA–DNA conjugates with RNAtr/DNA–Chol nanoparticles results in RNAtr/DNA–Chol/FA–DNA hybrids with the condensed nanostructures, on which folates direct outwards. A square bracket with subscript, N, indicates multiple tandem copies of RNA hairpins in the RNA transcripts. The box shows the simulated hairpin structure of single repeat unit of RNA transcripts. The small RNA hairpins in the DNA–Chol- or FA–DNA-binding region are an inevitable consequence of designing sequence.

First, we designed a linear single-stranded DNA (ssDNA) template to be complementary to each sequence of T7 promoter primer, sense anti-red fluorescent protein (RFP) siRNA, antisense anti-RFP siRNA, DNA–Chol conjugate and FA–DNA conjugate, as shown in Fig. 1 and Supplementary Table 1. The linear ssDNA, of which both ends are complementary to T7 promoter, was annealed with T7 promoter primer, yielding circular DNA with a nick. After DNA ligase connected the nicked sequence, T7 RNA polymerase generated the polymerized RNA transcripts (that is, RNAtr) from the closed circular DNA template via RCT. The RNA mfold program for RNA folding and hybridization prediction shows that the resulting RNA transcripts are composed of multiple tandem copies of hairpin RNA sequences, which can be converted to target siRNA by cytoplasmic Dicer after transfection.

**Figure 2:**
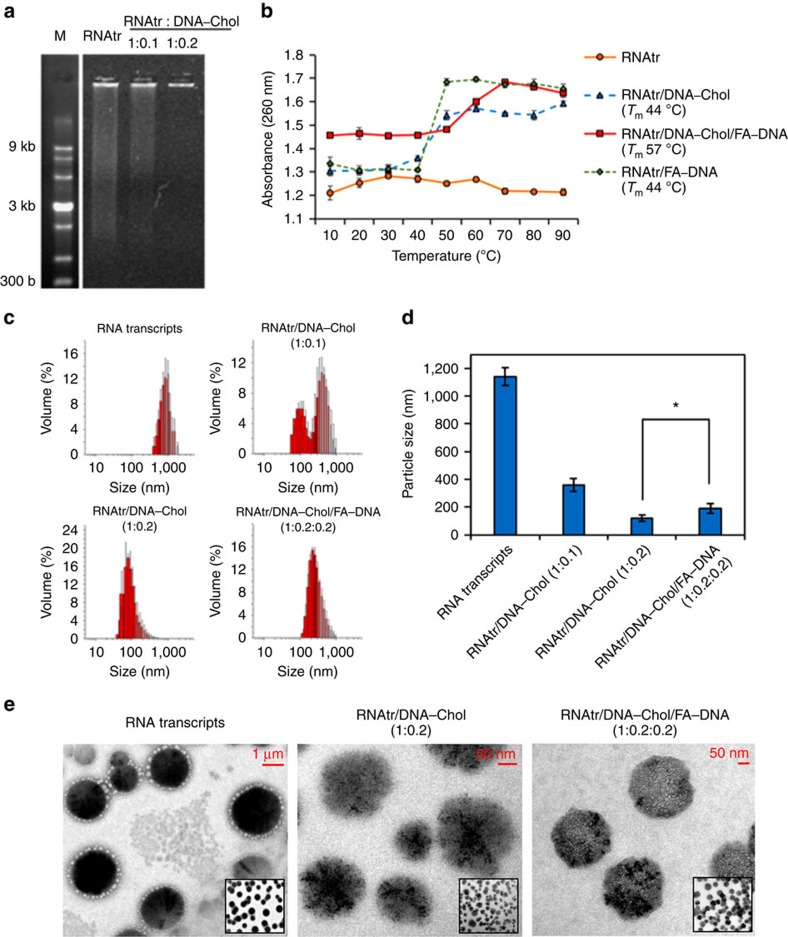
Characterization of RNA/DNA hybrids. (**a**) Gel retardation assay of RNA transcripts (RNAtr) and RNAtr/DNA–Chol hybrids at the indicated weight ratio. Nucleic acids were visible under ultraviolet irradiation after SYBR gold staining. (**b**) RNA/DNA thermal denaturation curves for RNA transcripts (RNAtr), RNAtr/DNA–Chol hybrids, RNAtr/FA–DNA hybrids and RNAtr NPs. *T*_m_ represents a transition from a RNA/DNA duplex to two strands. As temperature increased, each absorbance at 260 nm was plotted. (**c**,**d**) Size distribution profiles and average size of RNA transcripts, RNAtr/DNA–Chol hybrids, and RNAtr/DNA–Chol/FA–DNA hybrids at the indicated weight ratio. The results represent the mean±s.d. (*n*=5). *P<0.01 by one-way analysis of variance (ANOVA). *P<0.01 by one-way ANOVA. (**e**) Transmission electron microscopy images showing size and shape of particles. Insets show low-magnification images of particles.

Next, RNA transcripts were hybridized with DNA–Chol conjugates for self-assembly in the aqueous solution, because cholesterols have the property that becomes tightly packed under the limited space due to hydrophobic interactions^27^. The smeared bands observed on the gel electrophoresis demonstrated the polymerized RNA transcripts had a wide range of molecular weights, whereas the partially or fully hybridized RNAtr/DNA–Chol showed the different electrophoretic mobilities due to the self-assembled structures (Fig. 2a; Supplementary Fig. 1). At a weight ratio of 1:0.2, most RNAtr/DNA–Chol hybrids did not move down and stay in the well, although a maximum weight ratio is theoretically determined as 1:0.26. In addition, FA–DNA conjugates were hybridized with RNAtr/DNA–Chol hybrids to acquire a tumour-targeting ability. The resulting RNAtr/DNA–Chol/FA–DNA hybrids also showed the self-assembled structures with high molecular weights on the gel electrophoresis.

### Characterization of self-assembled RNA/DNA hybrids

We first examined the thermal stability of RNA/DNA hybrids. During thermal denaturation, RNAtr/DNA–Chol and RNAtr/FA–DNA hybrids had a melting temperature (*T*_m_) at 44 °C corresponding to a transition from a RNA/DNA duplex to two single strands (Fig. 2b). Particularly, RNAtr/DNA–Chol/FA–DNA hybrids revealed the significantly increased melting temperature (Δ*T*_m_=13 °C), which is closely associated with the stably hybridized structures. In the thermal denaturation profile of RNA transcripts alone, the small variations of absorbance around 20, 40 and 60 °C are probably the result of the presence of several different duplex species including the small hairpin structures (such as FA–DNA- or DNA–Chol-binding domains) as well as the major hairpin structures. Fortunately, these small variations of absorbance did not disturb the measurements of *T*_m_ in the RNA–DNA duplexes. When measured by dynamic light scattering, RNA transcripts had the micro-sized hydrodynamic diameter, ranging from 1.07 to 1.20 μm, but hybridization of RNA transcripts with DNA–Chol conjugates (1:0.2, w/w) significantly decreased their particle size down to 119.3±21.8 nm (Fig. 2c,d). A 10-fold decrease in size indicates that RNA/DNA hybrids self-assemble into highly packed nanoparticles due to the acquired amphiphilicity. The partially hybridized RNAtr/DNA–Chol, which formed at a weight ratio of 1:0.1, showed a relatively wide range of size distributions with two peaks, while their averaged size was estimated at 357.2±47.2 nm. The final product, RNAtr/DNA–Chol/FA–DNA hybrids (1:0.2:0.2, w/w/w) still showed the nano-sized particle size (190.1±37.2 nm). Furthermore, zeta potential, as measured by electrophoretic light scattering, showed the change of particle surface charge from −11.9±2.1 mV (RNA transcripts) to −1.5±3.3 mV (RNAtr/DNA–Chol/FA–DNA hybrids) in the presence of residual magnesium ions, which were supplemented during preparation steps to be finally 10 mM (Supplementary Fig. 2). Because magnesium ions compensate for the negative charge of oligonucleotide complexes^28^, it is anticipated that the shielding effect of magnesium ions will be more pronounced in the densely packed RNAtr/DNA–Chol/FA–DNA hybrids than in the RNA transcripts, due to the higher surface density. Transmission electron microscopy images also showed that RNAtr/DNA–Chol/FA–DNA hybrids, of which the shapes were spherical, had significantly smaller size than RNA transcripts and thus formed the nano-sized particles (RNAtr NPs) (Fig. 2e). Molecular weight of RNAtr NPs was estimated about 1,400 kDa, based on static light scattering (Supplementary Fig. 3). Using pyrene as a hydrophobic fluorescent probe, a critical micelle concentration of RNA transcripts was determined 0.6 μg ml^−1^ (Supplementary Fig. 4).

### Enhanced stability of RNAtr NPs

**Figure 3:**
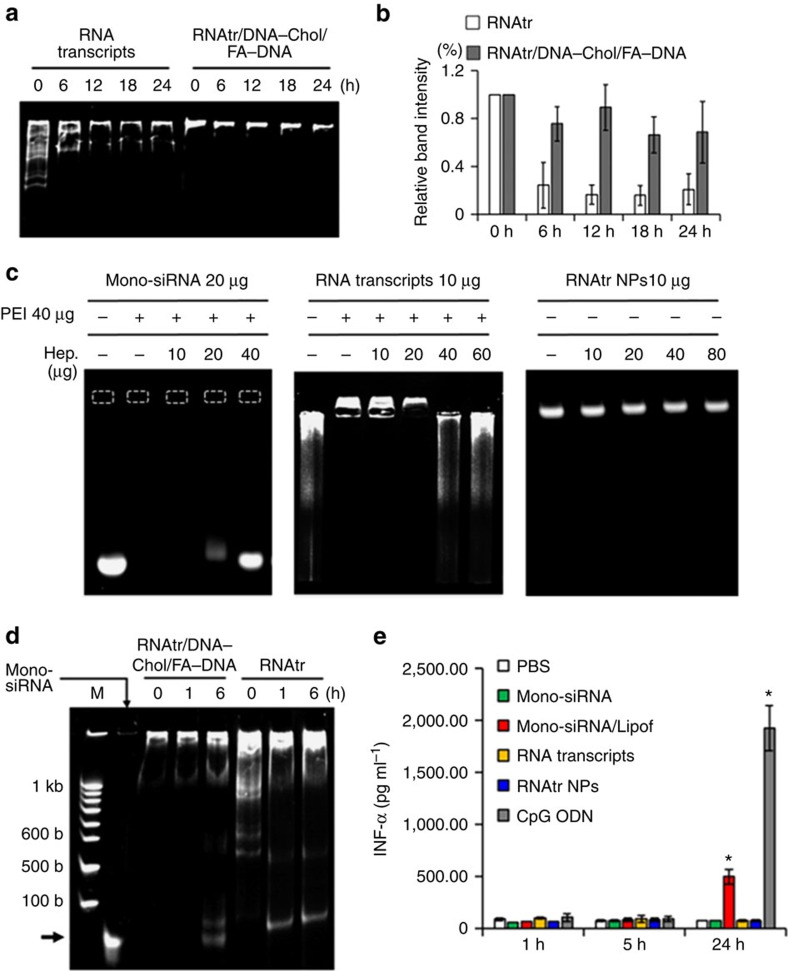
Stability and dicer cleavage experiments of RNAtr NPs. (**a**,**b**) Stability test under fetal bovine serum (FBS) condition. RNA transcripts and RNAtr NPs incubated in 30% FBS solution for the indicated time were analysed by non-denaturing polyacrylamide gel (15%) under TBE running buffer. The relative band intensities are plotted versus incubation time (**b**). The results represent the mean±s.d. (*n*=3). (**c**) Polyanionic heparin competition studies of siRNA/PEI complexes, RNA transcripts/PEI complexes and RNAtr NPs. siRNA/PEI complexes (20/40 μg), RNA transcripts/PEI complexes (10/40 μg) and RNAtr NPs (10 μg) were incubated with the various concentrations of heparin, and then the agarose gel (3%) electrophoresis was performed. The dashed box indicates a well. Remarkably, RNAtr NPs did not show the disassembled behaviours on agarose gel even under an eightfold weight excess of heparin. (**d**) Generating siRNA from RNA particles by Dicer cleavage *in vitro*. Four microgram RNA transcripts or 4 μg RNAtr NPs were reacted with 1.5 unit of recombinant human Dicer enzyme for the indicated time, and then electrophoresed on 3% agarose gel. The amount of generated short RNA strands from RNA transcripts or RNAtr NPs was measured relative to monomeric anti-RFP siRNA duplex (1 μg). M indicates the RNA ladder lane. (**e**) INF-α induction studies in the human peripheral blood mononuclear cells. INF-α induction was analysed 1, 5 and 24 h after incubation with mock (PBS), monomeric siRNA (100 nM siRNA), monomeric siRNA/lipofectamine complexes (equivalent to 100 nM siRNA), RNA transcripts (6 μg ml^−1^), or RNAtr NPs (9.65 μg ml^−1^). CpG oligodeoxynucleotides (5 μM) were treated as the positive control for INF-α induction. The results represent the mean±s.d. (*n*=3). *P<0.001 by one-way analysis of variance with Tukey's multiple comparison test, as compared with the PBS control.

One of major challenges for systemic delivery of siRNA is to prolong their stability against nuclease attack in the bloodstream^29^. When serum stability was examined on 15% non-denaturing polyacrylamide gel after 30% fetal bovine serum (FBS) treatment, RNA transcripts were readily degraded and about 20% remained after 24-h incubation, whereas RNAtr NPs were more resistant to nuclease attack and about 65% still remained even after 24-h incubation (Fig. 3a,b). In contrast, the naked monomeric siRNA were fully broken down within 1-h incubation (data not shown). These results indicate that RNAtr NPs would be stable from nuclease attack in the bloodstream due to their condensed structures. The additional analysis using a lower percentage polyacrylamide gel (5%), on which RNAtr NPs were not stuck in the wells but moved down, also supported their enhanced stability properties against serum nucleases (Supplementary Fig. 5). Furthermore, stability of RNAtr NPs against large polyanions was investigated through heparin competition studies. Polycationic polymers/siRNA complexes formed by charge–charge interactions might encounter unwanted exchange of siRNA with large polyanions (for example, sulfated glycosaminoglycans) outside cells, resulting in low siRNA delivery efficiency^16^. When incubated with heparin, monomeric siRNA/PEI complexes and RNAtr/PEI complexes were readily dissociated to each component (Fig. 3c; Supplementary Fig. 6). However, RNAtr NPs maintained their self-assembly even after incubation with an eightfold weight excess of heparin, because their condensation was formed by hydrophobic interactions, not by electrostatic interactions. These results indicate that stability of RNAtr NPs would be less affected by large polyanions in the extracellular matrix environment.

### Conversion of RNA hairpins into siRNA by Dicer

According to our delivery strategy, RNAtr NPs protect siRNA duplexes as hairpin structures during transport to the cytoplasm, but after transfection, it is necessary to convert RNA hairpins into siRNA duplexes by Dicer enzyme^2^. When incubated with Dicer, 4 μg RNAtr NPs were converted to 0.52 μg short RNA strands 6 h post reaction, whereas 4 μg RNA transcripts were converted to 0.84 μg short RNA strands 1 h post reaction (Fig. 3d). On the basis of our calculation, the theoretical loading efficacy (w/w) of siRNA duplex in RNAtr NPs is 24.4%, which means a quarter of weight of RNAtr NPs is occupied by siRNA duplexes (see Methods). Thus, 53.3% of theoretically cleavable siRNA in the RNAtr NPs are converted to short siRNA duplexes, and these experimental results mean that 13% (w/w) of RNAtr NPs are converted to short siRNA duplex. Compared with RNA transcripts, RNAtr NPs showed the different cleavage patterns including more than one repeat units, and required more reaction time to generate short siRNA duplexes, because RNAtr NPs have more highly packed structures.

### Immunogenicity studies

After systemic administration or in primary human blood cell cultures, it is reported that exogenous siRNA or siRNA/carriers induce high levels of inflammatory cytokines and interferons associated with innate immunogenicity^30^,^31^. When we measured interferon-α (INF-α) release using human peripheral blood mononuclear cells (PBMCs), CpG oligodeoxynucleotide, a positive inducer for INF-α, highly increased INF-α level 24 h post treatment, but neither RNAtr NPs nor RNA transcripts activated INF-α induction above basal level (Fig. 3e). Meanwhile, siRNA/Lipofectamine (equivalent to 100 nM siRNA) caused the significant secretion of INF-α possibly due to the immunostimulative activity of Lipofectamine. Because measurement of the immune response at an appropriate time after siRNA treatment is critical to make a valid assessment, we also measured INF-α release at the early time points including 1 and 5 h post treatment, but there was no detectable INF-α. Similarly, neither RNAtr NPs nor RNA transcripts caused any severe tumour necrosis factor-α (TNF-α) induction 1, 5 and 24 h post treatment, respectively, whereas lipopolysaccharide treatment lead to the remarkable elevation of TNF-α in the culture supernatant 5 and 24 h post treatment (Supplementary Fig. 7). These results suggest that RNAtr NPs have great potential in clinical use, because they do not trigger innate immune response. Furthermore, RNAtr NPs did not show any significant cytotoxicity on cell viability studies, up to the concentration of 50 μg ml^−1^ (Supplementary Fig. 8).

### Folate receptor-specific cellular binding and uptake

**Figure 4:**
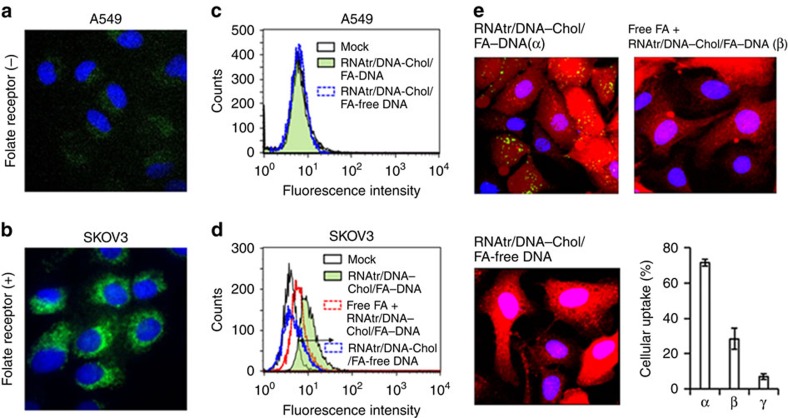
Cellular binding and uptake of RNAtr NPs. (**a**,**b**) Folate receptor-specific cell binding of RNAtr NPs in folate receptor-negative A549 lung adenocarcinoma cells and folate receptor-positive SKOV3 ovarian cancer cells. The fluorescence images were obtained by a fluorescence microscope with a CCD camera. Green and blue signals represent the fluorescence FAM-labelled RNAtr NPs and 4,6-diamidino-2-phenylindole (DAPI) dyes, respectively. (**c**,**d**) Flow cytometry studies of RNAtr NPs and folate-free RNAtr NPs. A prefixed gate region for FAM fluorescence is represented and the percentage of cells sorted within the gate is as follows; 8% (mock treatment), 82% (RNAtr NPs treatment), 11.6% (folate-free RNAtr NPs treatment) and 40% (folate preincubation before RNAtr NPs treatment). Noticeably, when folate receptors were saturated with an excess of folate ligands before RNAtr NPs treatment, the cell binding of RNAtr NPs was significantly reduced. These results indicate that RNAtr NPs can bind the cancer cells in a folate receptor-specific manner. (**e**) Intracellular uptake studies using confocal microscopic images. For clarity, the FAM-labelled RNAtr NPs and FAM-labelled folate-free RNAtr NPs were treated to RFP-expressing SKOV3 (SKOV3-RFP) cells. Red, green and blue signals represent the fluorescence RFP, FAM-labelled RNAtr NPs and DAPI dyes, respectively. When folate receptors were saturated with folate ligands, the cellular uptake of RNAtr NPs was significantly reduced. Quantification of the nanoparticles cellular uptake represents the mean±s.d. (*n*=3). For the image quantification, LAS AF Lite Software was used.

To examine the folate receptor-specific cell binding, fluorescein amidite (FAM)-labelled RNAtr NPs were transfected to folate receptor-positive SKOV3 ovarian cancer cells and folate receptor-negative A549 lung adenocarcinoma cells^32^,^33^. At 3 h post treatment, the fluorescence microscopic images exhibited the numerous punctate spots corresponding to RNAtr NPs within the SKOV3 cells, whereas there was relatively weak FAM signal within the A549 cells due to lack of folate receptor (Fig. 4a,b). Also, the flow cytometry showed that RNAtr NPs had about 82% cell binding efficiency in the SKOV3 cells 3 h post treatment, but folate-free RNAtr NPs showed only 11.6% cell binding efficiency due to the lack of folate ligands (Fig. 4c,d). Either RNAtr NPs or folate-free RNAtr NPs did not bind to the A549 cells due to the lack of folate receptor. When compared with FAM intensities within the SKOV3 cells on the fluorescence microscopic images, very low FAM intensities within the A549 cells may be due to nonspecific binding of RNAtr NPs to cell surface or endocytosis in a nonspecific manner such as macropinocytosis^34^, although the flow cytometry showed no detectable difference between RNAtr NPs-treated A549 cells and control A549 cells. Once the folate receptors present in SKOV3 cells were saturated with their ligands, the uptake efficiency of RNAtr NPs decreased to 40% on the flow cytometry studies. On the other hand, RNA transcripts were hardly transfected to SKOV3 cells possibly due to their strong negative charge and lack of folate (data not shown). In the confocal microscopic images of RFP-expressing SKOV3 (SKOV3-RFP) cells, RNAtr NPs were readily transfected into the cells with a cellular uptake efficiency of 71.7%, whereas their cellular uptake decreased to 28.3% in the folate-pretreated cells (Fig. 4e). The cellular uptake efficiency of folate-free RNAtr NPs also decreased to 7.0%. Taken together, these results indicated that RNAtr NPs can be internalized into cancer cells in a folate receptor-specific manner.

### Gene silencing effect *in vitro*

**Figure 5:**
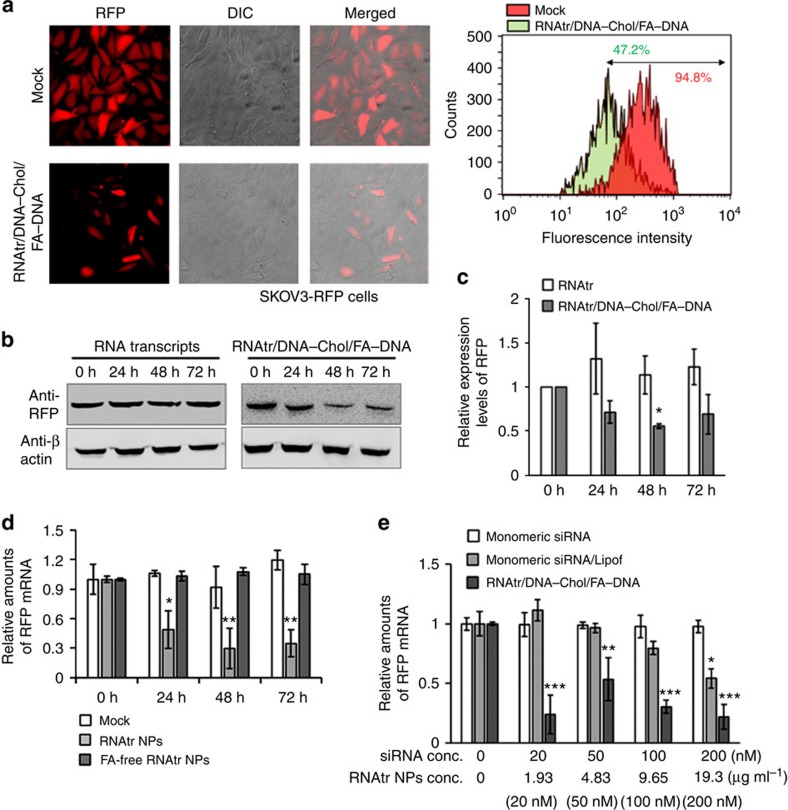
Silencing of target RFP gene in RFP-expressing SKOV3 cells. (**a**) Fluorescence microscopic images and flow cytometry analysis of RNAtr NPs-treated SKOV3-RFP cells. RNAtr nanoparticles were added to the cell culture media at the final concentration of 10 μg ml^−1^ (equivalent to 100 nM siRNA) and their gene silencing effect was measured 48 h post treatment. As a control, SKOV3-RFP cells only treated with PBS buffer were compared. The percentage number in the fluorescence-activated cell sorting profile represents the percentage of RFP-expressing cells sorted within a prefixed gate region. (**b**,**c**) Suppressed RFP expression measured by western blotting. The amount of RFP protein was measured in SKOV3-RFP cells treated with RNA transcripts or RNAtr nanoparticles, at the final concentration of 10 μg ml^−1^ for the indicated period. Relative expression levels of RFP were plotted versus incubation time (**c**). The results represent the mean±s.d. (*n*=3). *P<0.05 by one-way analysis of variance with Tukey's multiple comparison test, as compared with the corresponding control. (**d**) RFP mRNA measured by qRT–PCR. After treating SKOV3-RFP cells with either RNAtr NPs or folate-free RNAtr NPs at the final concentration of 10 μg ml^−1^ for the indicated periods, amounts of mRNA present in each sample were measured by qRT–PCR. The amounts of amplified RFP DNA were normalized against those of amplified β-actin DNA. (**e**) Silencing effect comparison using qRT–PCR. Suppressed RFP mRNA after treatment with monomeric anti-RFP siRNA, monomeric anti-RFP siRNA/Lipofectamine complexes and RNAtr nanoparticles was measured 48 h post treatment by qRT–PCR. For comparison with siRNA/Lipofectamine, the same amount of siRNA was theoretically generated from RNAtr nanoparticles at the concentration in parenthesis (see Methods). The results represent the mean±s.d. (*n*=3 for one of three independent experiments) (**d**,**e**). *P<0.05, **P<0.005, ***P<0.001 by one-way analysis of variance with Tukey's multiple comparison test, as compared with the corresponding controls.

The *in vitro* gene silencing of RNAtr NPs was examined by measuring RFP signals in RFP-expressing SKOV3 cells after transfection. The fluorescence microscopic images of RNAtr NPs-treated SKOV3-RFP cells showed that the RFP signals significantly decreased 48 h post treatment, whereas the control cells retained the intense RFP signals (Fig. 5a). The flow cytometry also demonstrated the RFP signals of RNAtr NPs-treated cells decreased down to 48%, compared with those of control cells. Consistently, the western blot showed that the RFP expression decreased down to 55% 48 h post treatment (Fig. 5b,c). Noticeably, RNA transcripts hardly suppressed RFP expression in SKOV3-RFP cells, because they could not be transfected to the cells. When the amounts of RFP messenger RNA (mRNA) remained in RNAtr NPs-treated cells were measured by quantitative PCR with reverse transcription (qRT–PCR), they significantly decreased down to 25% 48 h post treatment (Fig. 5d). The folate-free RNAtr NPs-treated cells, as a control, did not show any decrease of amounts of RFP mRNA at the indicated incubation times, because the folate-free RNAtr NPs were hardly transfected to the cells due to the lack of folate ligands. Next, we compared gene silencing efficiency of RNAtr NPs with that of Lipofectamine. The qRT–PCR analysis, measured 48 h post treatment, demonstrated that RNAtr NPs had better silencing efficiency than monomeric siRNA/Lipofectamine complexes, and even at the low concentration such as 1.93 μg ml^−1^ (equivalent to 20 nM siRNA), they significantly decreased the amount of RFP mRNA (Fig. 5e).

### Biodistribution in tumour model and gene silencing *in vivo*

**Figure 6:**
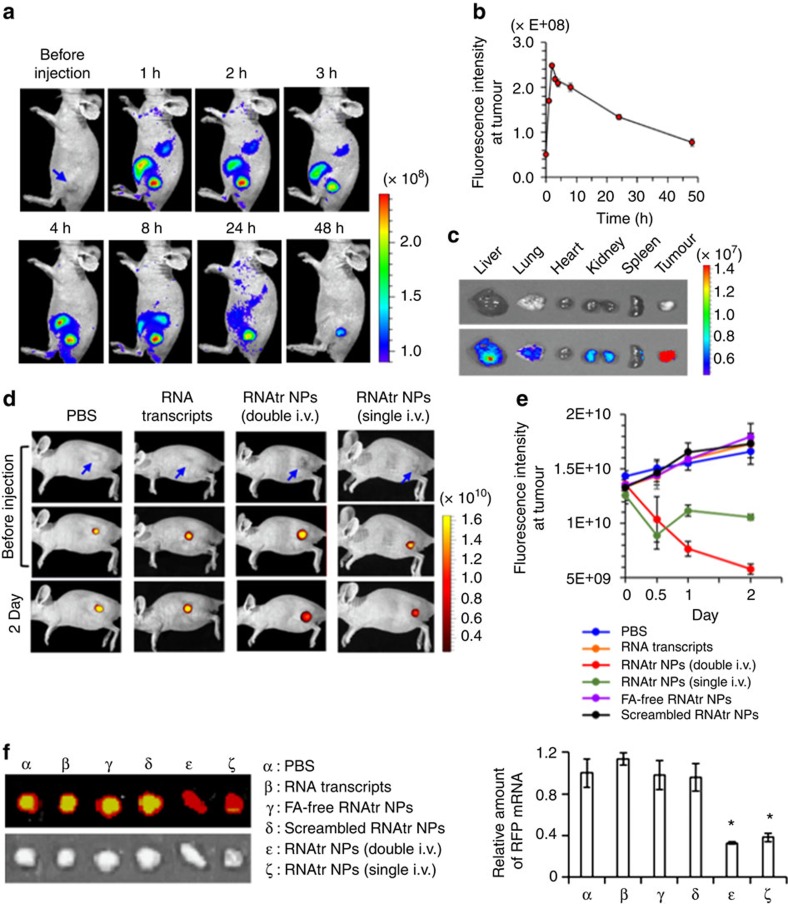
Biodistribution of RNAtr NPs and their RFP gene silencing in tumour model. (**a**,**b**) Whole-body NIRF imaging of SKOV3 tumour-bearing mice after intravenous injection of Cy5-labelled RNAtr NPs via tail vein. Blue arrow indicates the location of tumour xenograft. Fluorescence intensity of Cy5-labelled RNAtr NPs, which accumulated at tumour sites, was measured at the indicated time point (**b**). The results represent the mean±s.d. (*n*=4 mice). (**c**) *Ex vivo* NIRF images of the dissected organs and tumor tissues 48 h post injection. (**d**) Noninvasive NIRF imaging of *in vivo* RFP gene silencing from RNAtr NP-injected mice. Blue arrow represents site of tumour. RNAtr NPs were intravenously injected into the RFP-expressing SKOV3-RFP tumour-bearing mice via tail vein at an interval of one day (days 0, 1; 25 μg per injection) or only once (day 0; 50 μg). As the controls, either PBS or RNA transcripts were intravenously injected via tail vein once (day 0; 50 μg). Also, either folate-free RNAtr NPs or scrambled RNAtr NPs were intravenously injected via tail vein once (day 0; 50 μg). These results are shown in the Supplementary Fig. 10. (**e**) RFP fluorescence intensities at tumour sites measured before or after systemic administration were plotted versus day. The results represent the mean±s.d. (*n*=3 mice per group). (**f**) *Ex vivo* NIRF images of the dissected tumours from PBS-, RNA transcripts-, folate-free RNAtr NP-, scrambled RNAtr NP-, RNAtr NPs double-injected or RNAtr NPs single-injected mice. The tumours were excised 2 days post injection. Amount of RFP mRNA present in the dissected tumours was measured by qRT–PCR (right). The results represent the mean±s.d. (*n*=3 mice). Results represent one of three independent experiments (**f**, right). *P<0.001 by one-way analysis of variance with Tukey's multiple comparison test, as compared with the PBS control.

To verify *in vivo* tumour-targeting ability, Cy5-labelled RNAtr NPs (50 μg) were systemically administered into mice bearing SKOV3 xenograft tumours via tail vein, and their biodistribution was monitored by measuring near-infrared fluorescence (NIRF) images (Fig. 6a,b). When the weights of mice ranged from 20 to 25 mg, 50 μg of Cy5-labelled RNAtr NPs corresponded to a dose of 0.61 mg kg^−1^ of siRNA. At 1 h post injection, a strong NIRF signal observed in tumours clearly distinguished tumour tissues from other organs. The NIRF signal in tumours reached the maximum intensity 2 h post injection and persisted at least for 48 h. *Ex vivo* fluorescence images observed 48 h post-injection also corresponded well with the biodistribution images, and showed that RNAtr NPs accumulated primarily in tumours, compared with other dissected organs (Fig. 6c). The *ex vivo* images obtained at relatively earlier time point showed that RNAtr NPs already accumulated at tumours 1 h post-injection, although non-negligible quantities of RNAtr NPs were observed in the liver and kidney organs (Supplementary Fig. 9).

The *in vivo* gene silencing of RNAtr NPs was evaluated by RFP fluorescence images in the SKOV3-RFP xenograft tumours after systemic administration. When the fluorescence signals of RFP-expressing tumours were strongly detected, RNAtr NPs were intravenously injected into mouse once (day 0; 50 μg) or at an interval of 1 day (days 0, 1; 25 μg per injection), and then RFP signals at the tumour sites were measured (Fig. 6d,e; Supplementary Fig. 10). In 2 days post injection, the RFP signals decreased down to 45 and 80% in the RNAtr NPs double-injected and RNAtr NPs single-injected mice, respectively, when compared with those in the PBS-injected mice. However, each of RNA transcript-injected and folate-free RNAtr NP-injected mice revealed not decreased, but rather increased RFP signals at the tumours, because these nanoparticles could not accumulate at the tumours due to the lack of folate-targeting moiety. The scrambled RNAtr NP-injected mice showed the increase of RFP signals at the tumours, as expected. *Ex vivo* RFP signals of the tumours 2 days post injection, as well as the levels of RFP mRNA at the tumours, corresponded well with the *in vivo* real-time fluorescence images (Fig. 6f). Finally, the immune response of RNAtr NPs was evaluated by measuring INF-α and TNF-α levels in blood samples 1, 5 and 24 h post injection, respectively (Supplementary Fig. 11). Intravenous administration of siRNA/Lipofectamine lead to elevations of INF-α in serum by 5 h and resolution by 24 h after a single treatment. Compared with INF-α levels in the untreated mice, there was no significant increase 1, 5 and 24 h post injection. siRNA/Lipofectamine resulted in a similar pattern of TNF-α release, with the elevations in serum 1 and 5 h post injection, and the resolution 24 h post injection. However, RNAtr NPs did not lead to detectable TNF-α release at the indicated time points.

## Discussion

This study has demonstrated that our design of RNAtr NPs facilitates precise control over condensation of RNA polymers and the locations of folate ligands, through a combination of RCT and Watson–Crick base pairing. Finally, RNAtr NPs showed robust RFP gene silencing effects in tumour sites when intravenously injected into SKOV3-RFP tumour xenograft mice. Up to date, enzymatic RCT reaction has not been widely explored in the siRNA therapeutic applications, although there is great merit that it can generate RNA polymer in multiple copy numbers, at a much lower cost than using chemically synthesized RNA. Considering the high cost of therapeutic siRNA, our design of RNAtr NPs has great potentials as a platform technology in that enzymatic RCT reaction can be directly adopted for the targeted and systemic siRNA delivery.

An efficient condensation of siRNA can protect its degradation before cellular uptake as well as increase loading efficiency, and thus there have been extensive studies to increase siRNA's size, charge and flexibility^16^,^17^,^18^,^19^. However, these approaches have the difficulties to inevitably incorporate synthetic polycationic reagents, which lead to potentially harmful cytotoxicity. In contrast, RNAtr NPs provide an alternative means of condensing large amounts of multimeric RNA polymers into the compact nanoparticles using hydrophobic interactions, especially without the aid of synthetic polycationic reagents, and consequently boost the loading amount of siRNA up to 24.4% (w/w) of RNAtr NPs. Because the amounts of delivered siRNA to target tissues *in vivo* depend on the cargo capacity, our RNAtr NPs with highly loaded siRNA could lead to robust gene silencing in the tumour xenograft mice model without any detectable immune responses. Furthermore, our condensation approach via hydrophobicity, unlike polycations-wrapped siRNA, did not show unwanted exchange with large polyanions found in the extracellular matrix. Thus, our design of RNAtr NPs reduces the risk of immunogenicity and cytotoxicity by avoiding the use of synthetic polycationic reagents, and overcomes the critical problems found in the conventional delivery systems.

Certain siRNAs stimulate release of proinflammatory cytokines via TLR7 and TLR8 in a sequence-dependent manner^35^. In these two pathways, some sequence motifs such as 5′-UGUGU-3′ play important roles^36^. Some siRNAs can activate innate immunity by inducing interferon expression via PKR and TLR3 signalling pathways in a sequence-dependent manner^37^. Chemical modifications such as substitutions of 2′-*O*-methyl or 2′-fluoropyrimidines in the ribose can abrogate activation of Toll-like receptors and prevent toxicities caused by activation of type-I interferon pathway, while they do not compromise siRNA activity^38^. Our design of RNAtr NPs has the potential merit that can incorporate the chemical modifications such as 2′-*O*-methylation into RNA hairpin repeats by adding 2′-*O*-methyl NTP during RCT. In the current studies, the lack of innate immune response has been demonstrated in the absence of chemical modification, but the RNAtr NPs including chemically modified ribonucleotides would be able to avoid the unwanted toxicities of innate immune responses expected in a wide range of clinical applications.

Typically, to capitalize on the unique structural features of tumour vasculature, drug carriers such as liposomes and polymeric nanoparticles with an average size of 200 nm or less are ideal for EPR (enhanced permeability and retention) effect^39^, although other studies require narrower size range from ∼10–100 nm: nanoparticles smaller than 10 nm are rapidly cleared by the kidneys and larger nanoparticles (∼100–200 nm) are cleared by reticuloendothelial system in the liver^40^. Considering the average size of RNAtr NPs, their size is slightly larger than the commonly defined threshold of 100 nm, and therefore it could be partially responsible for nonspecific accumulation in the liver (Fig. 6a,c; Supplementary Fig. 9), although several particle characteristics such as size, shape and surface features govern the biodistribution profile of drug carriers^40^. Folate-complexed oligonucleotide nanoparticles, which have a hydrodynamic diameter of ∼28.6 nm as a results of programmable self-assembly of DNA and siRNA, also showed slight accumulation in healthy organs such as the liver, spleen, lung and heart, although they accumulated primarily in the tumour and kidney^41^.

The recent findings on the composition of RNA microsponge-like particles (RNAi MS)^19^ have reported that the RNAi MS particles are composite materials consisting of RNA and crystalline magnesium pyrophosphate sheets^42^. These studies demonstrated that RNAi MS particles were broken down with EDTA and the released RNA was completely degraded by RNase. In contrast, EDTA did not affect the RNase protection assays of RNAtr NPs when it is added before RNase treatment (data not shown), because the condensation of RNAtr NPs is driven by the hydrophobic interactions that occur during self-assembly, not by formation of magnesium pyrophosphate sheets.

RNAtr NPs are particularly suitable with respect to ease of large-scale production, and there is no problem in reproducibility that arises as the total volume of a reaction is increased. Also, a simple purification process of the nanoparticles, as one of several advantages, would be compatible with scale-up processes. On the other hand, analytical issues including *in vivo* pharmacokinetics profiles and off-target effects of siRNA should be assessed before the therapeutic applications of RNAtr NPs.

A variety of targeting ligands including Arg–Gly–Asp (RGD) peptides^43^ and aptamers^44^ have been formulated into the carriers for tumour-specific cargo delivery. The ease of ligand–DNA conjugation, when combined with our simple design for coating the targeting ligands, would facilitate the enhanced versatility for various cancers including leukaemias as well as solid tumours. Therefore, our design of RNAtr NPs, as a platform technology, provides a novel method to systemically deliver siRNA to tumour sites, and can be readily extended to therapeutic siRNA applications for broad cancer treatments.

## Methods

### Materials

Linear ssDNA template, T7 promoter primer, 3′-cholesterol-modified DNA conjugate (DNA–Chol), 5′-amine-modified DNA fragment for preparation of FA–DNA, 3′-Cy5-labelled and 5′-amine-modified DNA fragment for preparation of FA–DNA-Cy5, primers for qRT–PCR, scrambled sense siRNA, scrambled antisense siRNA and monomeric siRNA were all synthesized from Bioneer Co. (Daejeon, Korea). All DNA sequences are summarized in Supplementary Table 1. 1-Ethyl-3-(3-dimethylaminopropyl)carbodiimide (EDC), Sulfo-NHS and folate were purchased from Sigma (St Louis, MO, USA). Lipofectamine2000 was purchased from Invitrogen (USA). Cell culture media were obtained from WelGENE (Korea). The RNA mfold program (available in http://mfold.rna.albany.edu/?q=mfold/RNA-Folding-Form) was used to predict the RNA secondary structures^45^. Female BALB/c nude mice and female C57BL/6J mice were purchased from Orient Bio Inc. (Korea).

### Synthesis of polymerized RNA transcripts via RCT

After T7 promoter primers dissolved in nuclease-free water were added to linear ssDNA template solution (0.5 μM) at a molar ratio of 1:1, the mixture solution was denatured at 95 °C for 2 min and then slowly cooled down to 25 °C, yielding circular DNA/T7 promoter hybrids with the nick. We added T4 DNA ligase (Promega, USA) and ligation buffer (30 mM Tris-HCl (pH 7.8), 10 mM MgCl_2_, 10 mM DTT and 1 mM ATP) containing additional 5 mM ATP to the resulting hybrid solution, and connected the nick of circular DNA/T7 promoter hybrids at 16 °C for 12 h. The closed circular DNA/T7 promoter hybrids (0.5 μM) were mixed with T7 RNA polymerase (5 U μl^−1^) in the reaction buffer (4 mM Tris-HCl (pH 7.9), 10 mM MgCl_2_, 1 mM DTT and 0.2 mM spermidine) containing 2 mM rNTP (ribonucleotide solution mix, NEB) and 1 U μl^−1^ RNase inhibitor in the final concentration; the total reaction volume was 20 μl. The mixture was incubated at 37 °C for 1 h, and consequently the polymerized RNA transcripts could be generated. At the end of RCT reaction, DNase 1 (2 U μl^−1^, NEB) was treated for 37 °C for 15 min and then enzymes present in the reaction mixture were inactivated by heating at 90 °C. The concentration of prepared RNA transcripts was measured using Quant-iT RNA Assay Kit (Invitrogen).

### Preparation of folate–DNA conjugate

We added 5′-amine-modified DNA fragment (10 mM), which was dissolved in conjugation buffer (100 mM MES, 500 mM NaCl, pH 6.0), to Sulfo-NHS (10 mM) and EDC (4 mM) solution, and then reacted the resulting DNA mixture with a 10-fold molar excess of folates for 3 h at 22 °C. β-Mercaptoethanol was added for deactivation of unreacted EDC. The 5′-amine group of DNA fragment was conjugated with carboxylate group of folate via EDC/Sulfo-NHS coupling reaction. To remove the reagents of conjugation, we filtered the obtained FA–DNA conjugates using Amicon ultracentrifugal filter (3 K MWCO, Millipore) while repeatedly adding nuclease-free water. In a similar way, 5′-amine group of 3′-FAM-labelled DNA fragment, which was obtained from Bioneer Co., was conjugated with carboxylate group of folate, yielding the fluorescent FA–DNA–FAM conjugate. To quantify a ratio of folate to DNA on FA–DNA conjugates, extinction coefficients of pure folate and pure DNA in water were measured at two different wavelengths, 260 and 363 nm, using standard curves plotted at both wavelengths (ultraviolet–visible spectrophotometry, Agilent, USA). The ratio of folate to DNA on FA–DNA conjugates is determined by solving the simultaneous equations for the concentration of folate and the concentration of DNA as previously described^46^: *A*_260_=*ɛ*_260_(FA) × [FA]+*ɛ*_260_(DNA) × [DNA] and *A*_363_=*ɛ*_363_(FA) × [FA]+*ɛ*_363_(DNA) × [DNA]. When extinction coefficients of folate and DNA were calculated by measuring the absorbance at two different wavelengths, the molar ratio of folate to DNA on FA–DNA conjugates was determined 0.95±0.2.

### Formation of RNAtr/DNA–Chol/FA–DNA hybrids

First, we mixed DNA–Chol conjugates (1.43 μg, dissolved in nuclease-free water) with the RNA transcripts (7.14 μg, dissolved in the reaction buffer) at the indicated weight ratio of RNAtr/DNA–Chol (1:0.2, w/w), and supplemented additional MgCl_2_ in the final concentration of 10 mM until a final volume reaching 20 μl. The mixture solutions were heated to 65 °C for 5 min to denature RNA transcripts and then slowly cooled down to 4 °C for 2 h, yielding RNAtr/DNA–Chol hybrids. Next, we mixed FA–DNA conjugates (1.43 μg, dissolved in nuclease-free water) with the RNAtr/DNA–Chol hybrids (8.57 μg) at the indicated weight ratio of RNAtr/DNA–Chol/FA–DNA (1:0.2:0.2, w/w/w), and supplemented additional MgCl_2_ in the final concentration of 10 mM until a final volume reaching 20 μl, yielding RNAtr/DNA–Chol/FA–DNA hybrids. We purified the resulting RNAtr NPs using Amicon ultracentrifugal filter (3 K MWCO, Millipore) while repeatedly adding PBS buffer containing MgCl_2_. For synthesis of RNAtr/DNA–Chol/FA–DNA hybrids, the weight ratio of 1:0.2:0.2 was chosen throughout the current studies. In a similar way, we obtained folate-free RNAtr NPs by mixing folate-free DNA with the RNAtr/DNA–Chol hybrids at the same weight ratio. Using the FAM-labelled folate-free DNA (folate-free-DNA-FAM), we also prepared the RNAtr/DNA–Chol/folate-free-DNA-FAM nanoparticles for flow cytometry studies. Scrambled RNAtr NPs, which contain the scrambled RFP siRNA sequences, were generated by the same procedure as mentioned above.

### Gel retardation assay

RNA transcripts, RNAtr/DNA–Chol hybrids and RNAtr/DNA–Chol/FA–DNA hybrids at the indicated weight ratio were analysed on 3% agarose gel. To distinguish FA–DNA conjugates from DNA–Chol conjugates, we used the FAM-labelled FA–DNA conjugates (FA–DNA–FAM). Nucleotides bands were visible under ultraviolet irradiation after SYBR gold staining. Throughout the current studies, the gel images were obtained by a Gel Doc image analysis system (Bio-Rad, USA). To measure the FAM fluorescence bands, we used a 12 bit charge-coupled device camera (ISM 2000MM, Kodak, USA).

### Static light scattering

Molecular weight of RNA transcripts was measured by a Zetasizer Nano ZS (Malvern, UK) and obtained from a Debye plot using Zetasizer Nano software (Supplementary Fig. 3). On the basis of molecular weight of RNA transcripts (1,000±387 kDa), molecular weight of RNAtr/DNA–Chol/FA–DNA nanoparticles (1:0.2:0.2, w/w/w) was estimated 1,400±541.8 kDa.

### Thermal denaturation of RNAtr NPs

Immediately after temperature of samples was adjusted to that of heat block, we measured the absorbance of samples at 260 nm using ultraviolet–visible spectrophotometry (Agilent). As temperature increased, the thermal denaturation curves were plotted. After the melting data were smoothed using PeakFit Peak Separation and Analysis Software (v 4.12), the value of *T*_m_ was determined as the maximum of the fittest first derivative melting plot.

### Characterization of particles

We measured the hydrodynamic size and zeta potential of particles by a Zetasizer Nano ZS (Malvern, UK). The particles were diluted in nuclease-free water. The autocorrelator collected the scattered light at an angle of 173°. Each measured value was an average of at least five independent measurements and each of measurements composed of >11 data collections. A CM30 electron microscope (Philips, CA, USA) for transmission electron microscopy images was used to analyse the morphology and size of particles. The CM30 was operated at an acceleration voltage of 200 kV. A drop of sample solution was placed on a 200-mesh copper grid coated with carbon and then air-dried over 2 h at room temperature.

### *In vitro* stability test of RNAtr NPs under serum condition

FBS was mixed with 0.1 ml RNA transcripts (200 μg ml^−1^) or RNAtr NPs (200 μg ml^−1^) at the final concentration of 30% and then incubated at 37 °C during the indicated period. Each of samples were analysed on the 15% non-denaturing polyacrylamide gel (15%) under TBE buffer.

### Heparin polyanion competition assay

Twenty microgram monomeric siRNA was incubated with 40 μg PEI (branched, 25 kDa) for 15 min and then heparin sodium, ranging from zero to 40 μg, was incubated with siRNA/PEI complexes for 15 min. The dissociated siRNA from siRNA/PEI complexes was analysed on 3% agarose gel under TAE buffer. Next, RNA transcripts/PEI complexes (10 μg/40 μg) or RNAtr NPs (10 μg) were incubated with heparin sodium in a similar way, and then electrophoresed as well.

### *In vitro* Dicer cleavage assay

Twenty microlitre of RNA transcripts (200 μg ml^−1^) or RNAtr NPs (200 μg ml^−1^) was reacted with 1.5 unit of recombinant human Dicer enzyme (Genlantis, USA) under reaction buffer condition containing 10 mM ATP and 50 mM MgCl_2_. After the mixture was incubated at 37 °C during the indicated period, reaction was quenched by adding 2 μl Dicer Stop Solution (Genlantis). Each sample was analysed on 3% agarose gel under TAE buffer.

### Theoretical loading efficacy (w/w) of siRNA in RNAtr NPs

The loading efficacy of anti-RFP siRNA duplex in RNAtr NPs delivery systems is theoretically defined as follows. Molecular weights of 114 base RNA strands generated during one cycle of RCT=36571.7 g mol^−1^. Molecular weights of anti-RFP siRNA duplex=12,510.3 g mol^−1^. Molecular weights of DNA–Chol conjugate=18,626.8 g mol^−1^. Molecular weights of FA–DNA conjugate=18,233.7 g mol^−1^. Therefore, the theoretical loading efficacy (w/w) of siRNA duplex in RNAtr NPs (%)=weight of anti-RFP siRNA duplex/{weight of (114 base RNA transcripts)/DNA–Chol/FA–DNA hybrid (1:0.2:0.2, w/w/w)} × 100 (%)=(12,510.3 g mol^−1^)/(51,200.4 g mol^−1^) × 100 (%)=24.4 (%).

### Amount of generated siRNA from RNAtr NPs by Dicer *in vitro*

One microgram of anti-RFP siRNA duplex (equivalent to 19 bp) alone as a standard was loaded on gel electrophoresis as shown in the Fig. 3d, and then its band intensity was compared with that of short RNA generated from RNAtr NPs by Dicer cleavage *in vitro* following the previously reported methods^19^. The band intensities corresponding to either standard duplex or short RNA duplex were analysed by a Gel Doc image analysis system. On the basis of the band intensity of the known amount of RNA standard, the relative amount of short RNA generated from RNAtr NPs was estimated (Supplementary Table 2). All experiments were repeated as triplicate. According to our experiments, the recombinant Dicer enzyme converted 4 μg RNAtr NPs to 0.52 μg short RNA strands. Therefore, the theoretically cleavable siRNA of 4 μg RNAtr NPs=4 μg × 24.4%=0.976 μg. The fraction of experimentally cleaved siRNA in the theoretically cleavable siRNA=0.52/0.976 μg × 100=53.3%. Thus, 53.3% of theoretically cleavable siRNA in the RNAtr/DNA–Chol/FA–DNA hybrids was generated into short siRNA strands.

### Cell culture

SKOV3 and A549 cells were cultured in McCoy's 5A and Ham's F-12 supplemented with 10% FBS plus 100 μg ml^−1^ penicillin–streptomycin (WelGENE, Korea), respectively. SKOV3-RFP cells were cultured in DMEM plus 3 μg ml^−1^ of puromycin (InvivoGen, UK) supplemented with 10% FBS and 100 μg ml^−1^ penicillin–streptomycin. SKOV3 and A549 cells were purchased from Korean Cell Line Bank (KCLB, Korea) and SKOV3-RFP cells from Cell biolabs (USA).

### Cell viability studies

We seeded SKOV3 cells on 96-well plates at density of 2 × 10^3^ cells per well. The attached cells were incubated with serum-free media for 30 min before RNAtr NPs treatments. After adding 200 μl RNAtr NPs dissolved in serum-free media to each well in a dose-dependent manner, we further incubated the cells for 3 h. Next, we removed the cell media by aspiration and refilled fresh media supplemented with 10% FBS to each well by 200 μl. After 24 h post treatment, we added MTT solution to each well up to a final concentration of 0.05 mg ml^−1^, and then incubated the cells for 3.5 h. After collecting the solutions, we added 200 μl dimethylsulfoxide to each of collected solutions to dissolve purple formazan crystals. Absorbance was measured using a microplate reader (Spectra MAX 340, Molecular Devices, USA) at 570 nm.

### INF-α and TNF-α analyses

We first isolated human PBMCs from whole blood using a standard Ficoll-Paque density-gradient centrifugation as previously described^47^. We carefully layered 20 ml diluted blood over 10 ml Lymphoprep solution in a 50-ml conical tube. After centrifuging at 400*g* for 20 min at 18 °C, we aspirated the upper layer leaving the mononuclear cell layer undisturbed at the interphase, and carefully transferred the mononuclear cell layer to a new conical tube. After washing with PBS, the isolated PBMCs were seeded in RPMI 1640 supplemented with 10% FBS plus 2 mM L-glutamine into 96-well plates at density of 3 × 10^4^ cells per well. When attachment of the cells was completed, we activated the cells with a variety of stimuli: monomeric siRNA (100 nM), monomeric siRNA/Lipofectamine (equivalent to 100 nM siRNA), RNA transcripts (6 μg ml^−1^), RNAtr NPs (9.65 μg ml^−1^) and CpG oligodeoxynucleotides (5 μM) for INF-α or lipopolysaccharides (50 ng ml^−1^) for TNF-α. On the basis of theoretical calculation as mentioned above, the same amounts of siRNA were generated from RNA transcripts or RNAtr NPs. CpG oligodeoxynucleotides and lipopolysaccharides were used as a positive control of INF-α and TNF-α, respectively. At the indicated time points such as 1, 5 and 24 h post treatment, we collected the supernatants and quantified the released INF-α and TNF-α using VeriKine Human Interferon Alpha ELISA Kit (PBL Biomedical, USA) and sandwich ELISA kit (Abcam, USA), respectively. All experiments were repeated as triplicate.

To evaluate immune responses *in vivo*, plasma INF-α level was measured using female C57BL/6J mouse. Each of three mice was intravenously injected with a variety of stimuli: monomeric siRNA/Lipofectamine (equivalent to 200 nM siRNA) and RNAtr/DNA–Chol/FA–DNA nanoparticles (50 μg) in a 200 μl injection volume. Control mice were injected with PBS solution. After 1, 5 and 24 h injection, respectively, blood samples were harvested from the mice and plasma INF-α levels were quantified using mouse INF-α-Ab ELISA kit (eBioscience, USA) according to the manufacturer's instruction. Similarly, plasma TNF-α level was monitored using mouse TNF-α-Ab ELISA kit (Thermo scientific, USA).

### Folate receptor-dependent cellular binding and uptake of RNAtr NPs

The fluorescent FAM-labelled RNAtr NPs (RNAtr/DNA–Chol/FA–DNA–FAM) were prepared as mentioned above. Each cell line of folate receptor-negative A549 and folate receptor-positive SKOV3 were plated on a confocal 35-mm clear coverglass-bottom Petri dish at a density of 1.5 × 10^5^ cells per well and grown until reaching about 70% confluency. After the cells were incubated in DMEM media for 30 min, the FAM-labelled RNAtr NPs were treated to them at the final concentration of 10 μg ml^−1^. At 3 h post treatment, the cells were washed three times with Dulbeccòs Phosphate Buffered Saline (DBPS), fixed with 4% paraformaldehyde-containing solution and stained with 4,6-diamidino-2-phenylindole. We observed the cellular uptake of FAM-labelled RNAtr NPs in A549 or SKOV3 cells using a fluorescence microscope (DeltaVision microscope, Olympus IX71, Japan) with an IX-HLSH100 Olympus camera. Also, the cellular uptake of FAM-labelled RNAtr NPs (10 μg ml^−1^), Cy5-labelled RNA transcripts (10 μg ml^−1^) or FAM-labelled folate-free RNAtr NPs (RNAtr/DNA–Chol/folate-free-DNA-FAM, 10 μg ml^−1^) was measured by flow cytometry (EasyCyte System, Guava Technologies, USA) using a blue laser (488 nm). To fluorescently label the RNA transcripts, Cyanine 5-dUTPs (Enzo, USA) were used during RCT reaction. For folate competition studies, we pretreated folates to SKOV3 cells at the final concentration of 1 mM for 30 min and subsequently treated the FAM-labelled RNAtr NPs at the final concentration of 10 μg ml^−1^. To assess the cellular uptake, confocal microscopic images were measured by a confocal laser scanning microscope (TCS SP8, Leica Microsystems, Germany). Leica TCS SP8 confocal system was equipped with 405-nm laser (for 4,6-diamidino-2-phenylindole), 488-nm laser (for FAM) and 514-nm laser (for RFP). Images obtained with a 40 × immersion objective were processed and quantified using LAS AF Lite software.

### Gene silencing of RFP gene in cell culture system

SKOV3-RFP cancer cells were plated at a density of 2 × 10^5^ cells per well on slideglass bottomed 35-mm culture dishes and grown to 70% confluency in DMEM media supplemented with 10% FBS plus antibiotics. After the cells were incubated in DMEM media supplemented with antibiotics for 30 min to maintain the folate-free condition, RNAtr NPs were added to the cell culture media at the final concentration of 10 μg ml^−1^. At 3 h post treatment, the cells were washed three times with DPBS and incubated in DMEM media containing 10% FBS plus antibiotics for 45 h. We thoroughly washed the cells with DPBS, fixed them with 4% paraformaldehyde-containing solution and then obtained the RFP fluorescence microscopic images. For the flow cytometry analysis, we followed the same procedure as described in the cellular uptake experiments.

### Western blotting

RNA transcripts or RNAtr NPs were treated to SKOV3-RFP cells at the final concentration of 10 μg ml^−1^ for the indicated period. To detect RFP proteins, each of cell lysates, prepared in RIPA buffer (Sigma, USA), were analysed using horseradish peroxidase-conjugated anti-RFP polyclonal antibody (Rockland, USA). The relative expression levels of RFP gene was normalized against expression of β-actin gene using anti-β-actin monoclonal antibody/anti-mouse IgG secondary antibody (Rockland). The western blots were developed with ECL solution (Bio-Rad) and their images were visualized by EZ-Capture MG (Japan).

### Comparison of RNAtr NPs with Lipofectamine

To compare silencing efficiency of RNAtr NPs with that of Lipofectamine, a conventional transfection reagent^48^, we theoretically calculated the amounts of RNAtr NPs needed to produce the same amounts of anti-RFP siRNA duplexes that Lipofectamine delivers (Fig. 5e). For example, 0.965 ng RNAtr NPs can generate 0.125 ng anti-RFP siRNA duplex (equivalent to 10 fmol). Monomeric anti-RFP siRNA/Lipofectamine complexes were produced at a 1:4 ratio (v/w) according to the manufacturer's instructions. Monomeric anti-RFP siRNA or monomeric anti-RFP siRNA/Lipofectamine complexes were added to the cell culture media up to 200 nM.

### Quantitative reverse transcription PCR

RNAtr NPs were treated to SKOV3-RFP cells at the final concentration of 10 μg ml^−1^ for the indicated period. mRNA present in each sample was isolated using RNeasy Mini Kit (Qiagen, USA) and then reverse-transcribed to complementary (cDNA) using High Capacity RNA-to-cDNA kit (Applied Biosystems, USA). Next, the resulting cDNA was mixed with 2 × SYBR Green master Mix (Applied Biosystems), RFP primer pair, or β-actin primer pair and then amplified by a StepOnePlus real-time PCR systems (Applied Biosystems). The amounts of amplified RFP DNA were normalized against those of amplified β-actin DNA.

### *In vivo* tumour-targeting studies of RNAtr NPs

For the real-time biodistribution imaging of RNAtr NPs, SKOV3 tumour cells (1 × 10^7^) cells were subcutaneously inoculated to the left flank of mouse (5-week-old female BALB/c nude mouse) and then grown until the tumour volume reached about 80 mm^3^. The fluorescently Cy5-labelled RNAtr NPs were prepared using 3′-Cy5-labelled FA–DNA conjugates and diluted in PBS. SKOV3-bearing nude mice were intravenously injected via tail vein with Cy5-labelled RNAtr NPs (2 mg kg^−1^, 200 μl per mouse; four mice). The whole-body images were obtained at the indicated time points (0–48 h after administration) using IVIS Spectrum (Caliper Life Science Inc., USA) and normalized using IVIS Living Imaging Software. Mice were killed 1 h post injection and 2 days post injection, respectively, to dissect tumours and organs including the liver, lung, spleen, kidney and heart, and then the *ex vivo* images were visualized using IVIS Spectrum. All animal care and experimental procedures were performed according to the regulations of the Institutional Animal Care and Use Committee of Korea Institute of Science and Technology.

### *In vivo* gene silencing studies

To investigate the *in vivo* RFP gene silencing of RNAtr NPs, 5-week-old female BALB/c nude mice were subcutaneously inoculated in the left flank with SKOV3-RFP cells (1 × 10^7^ cells per mouse). When the RFP-expressing tumours grew to 6–8 mm in diameter and their RFP fluorescence were strongly detected, RNAtr NPs were intravenously injected via tail vein at an interval of one day (days 0, 1; 25 μg per injection) or only once (day 0; 50 μg). To quantify RFP gene silencing, RFP signal intensities at tumours were monitored at the indicated time points (0–2 days after administration) using IVIS Spectrum and IVIS Living Imaging Software. As the controls, PBS or RNA transcripts (50 μg) were systemically administered once, and then the RFP fluorescence intensities of tumours were compared (*n*=3 for each group). Mice were killed 2 days post injection to excise tumours and the *ex vivo* images of tumours were obtained using IVIS Spectrum. To quantify the levels of RFP mRNA present in the excised tumours, the tumour samples were lysed and then homogenized. The RNA extraction and amplification steps followed the same protocols as described above. The *in vivo* gene silencing effects of folate-free RNAtr NPs and scrambled RNAtr NPs were examined in a similar way.

## Additional information

**How to cite this article:** Jang, M. *et al*. Design of a platform technology for systemic delivery of siRNA to tumours using rolling circle transcription. *Nat. Commun*. 6:7930 doi: 10.1038/ncomms8930 (2015).

## Supplementary Material

Supplementary Information Supplementary Figures 1-12 and Supplementary Tables 1-2
