# Supplementary material for: Design of a platform technology for systemic delivery of siRNA to tumours using rolling circle transcription
Source: Nat Commun. 2015 Aug 6;6:7930. doi: 10.1038/ncomms8930 (PMC4918333; doi:10.1038/ncomms8930)
Supplement: Supplementary Information — Supplementary Figures 1-12 and Supplementary Tables 1-2 [file ncomms8930-s1.pdf]

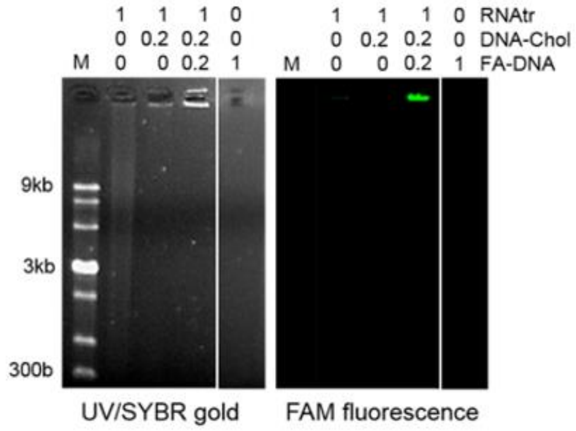

Supplementary Figure 1. Gel retardation assay of hybrids. Gel retardation assay showing formation of RNAtr/DNA-Chol/FA-DNA hybrids at the indicated weight ratio. To distinguish FA-DNA conjugates from DNA-Chol conjugates, the FAM-labeled FA-DNA conjugates (FA-DNA-fam) were used. Nucleic acids were visible under UV irradiation after SYBR gold staining, and the FAM fluorescence was measured by a 12 bit CCD camera. FA-DNA-fam conjugates alone moved fast and got off agarose gel, as shown in the last lane.

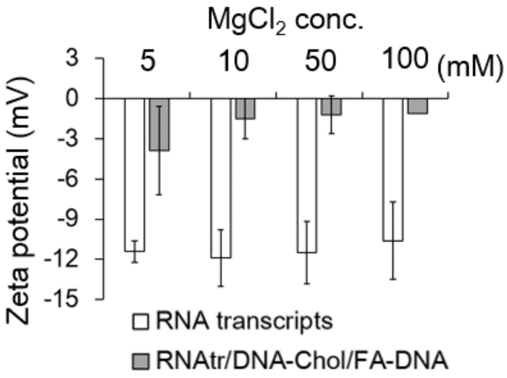

Supplementary Figure 2. Zeta potential of hybrids. Determination of zeta potential using electrophoretic light scattering (ELS). The particles were diluted in nuclease-free water containing magnesium ions at the indicated final concentration. The results represent the mean ± s.d. (n = 3).

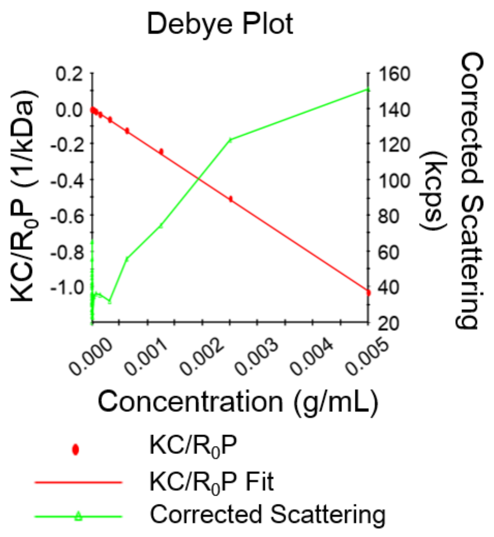

Supplementary Figure 3. A Debye plot of RNA transcripts from Static Light Scattering. Molecular weight of RNA transcripts was obtained from intercept at zero concentration. The theoretical MW of RNA strand generated during one cycle of RCT is 36571.7 g/mol, and when based on MW of RNA transcripts (1000 ± 387 kDa) estimated by static light scattering, the number of times the plasmid is transcribed in RCT reaction is theoretically calculated as 16.8 – 37.9.

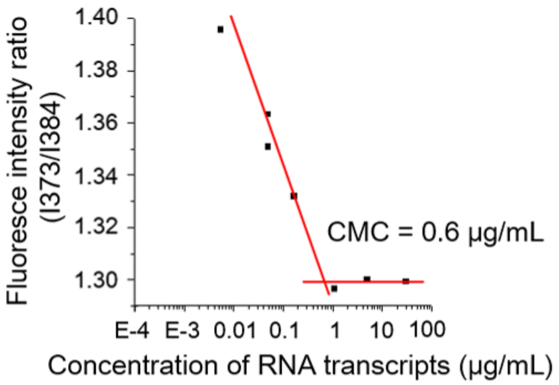

Supplementary Figure 4. Critical micelle concentration of RNA transcripts. 0.25 mM stock solution of pyrene in acetone was prepared and further diluted to give a final concentration of 6×10^{-7} M. Concentrations of RNA transcripts ranging from 5.0×10^{-3} μg/mL to 30 μg/mL were dissolved in a constant volume of water (1 mL). 50 μL of the diluted pyrene solution was added to each sample. The fluorescence was read using an excitation wavelength of 334 nm, with 5 nm of emission bandwidth. The emission spectra ranging from 350 nm to 450 nm were monitored using a LS-50 Fluorescence spectrometer (PerkinElmer, USA). The intensity ratios (I373/I384) of peaks at 373 nm to those at 384 nm of pyrene were plotted according to the concentrations of RNA transcripts. The ratio decreased with concentration forming a slope that was used to estimate the critical micelle concentration.

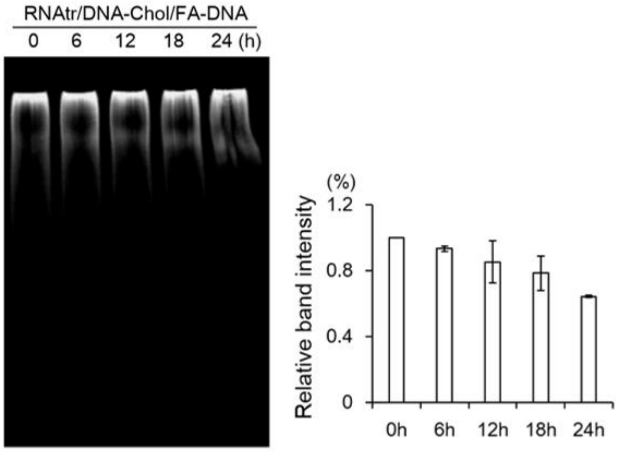

Supplementary Figure 5. Stability studies under FBS condition. RNAtr NPs were incubated in 30% FBS solution for the indicated times and then analyzed by non-denaturing polyacrylamide gel (5%) under TBE running buffer. The relative band intensities are plotted versus incubation time. The results represent the mean ± s.d. (n = 3). RNAtr NPs were stuck in the wells on 15% polyacrylamide gel as shown in the Fig. 3a, but moved down on 5% polyacrylamide gel. 5% polyacrylamide gel provided a more reliable tool to examine whether FBS degraded RNAtr NPs.

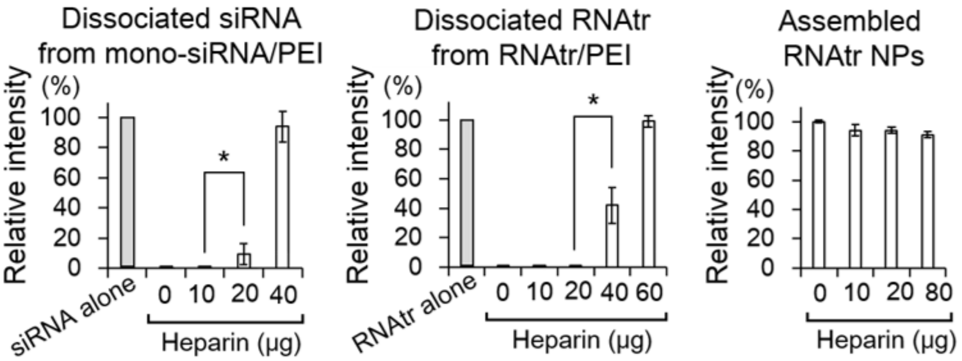

Supplementary Figure 6. Polyanionic heparin competition studies. Relative intensities of dissociated siRNA from monomeric siRNA/PEI complexes or dissociated RNA transcripts from RNA transcripts/PEI complexes, as shown in the Fig. 3c, are plotted versus amount of heparin. Also, relative intensity of self-assembled RNAtr NPs was plotted versus amount of heparin. The results represent the mean ± s.d. (n = 3). * $p < 0.001$ by one-way ANOVA.

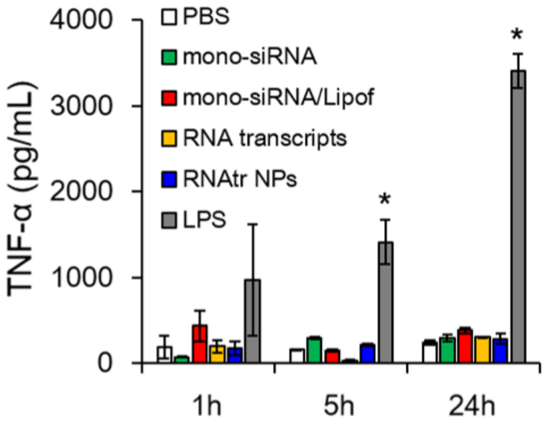

Supplementary Figure 7. TNF-α induction in human peripheral blood mononuclear cells. TNF-α induction was analyzed 1 h, 5 h and 24 h after incubation with mock (PBS), monomeric siRNA (100 nM siRNA), monomeric siRNA/lipofectamine complexes (equivalent to 100 nM siRNA), RNA transcripts (6 μg/mL), or RNAtr NPs (9.65 μg/mL). Lipopolysaccharides (50 ng/mL) were treated as the positive control for TNF-α induction. The results represent the mean ± s.d. (n = 3). * $p < 0.001$ by one-way ANOVA with Tukey`s multiple comparison test, as compared to the PBS control.

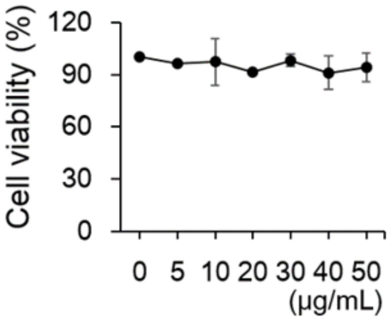

Supplementary Figure 8. Cell viability test of RNAtr NPs. *In vitro* cytotoxicities of SKOV3 cells treated with RNAtr NPs were measured by MTT assay. The results represent the mean ± s.d. (n = 3).

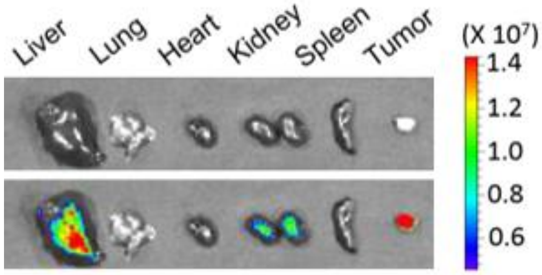

Supplementary Figure 9. *Ex vivo* NIRF images of the dissected organs and tumor tissues. Cy5-labeled RNAtr NPs were intravenously administered into SKOV3 tumor-bearing mice *via* tail-vein and the mice were sacrificed 1 h post-injection for *ex vivo* images.

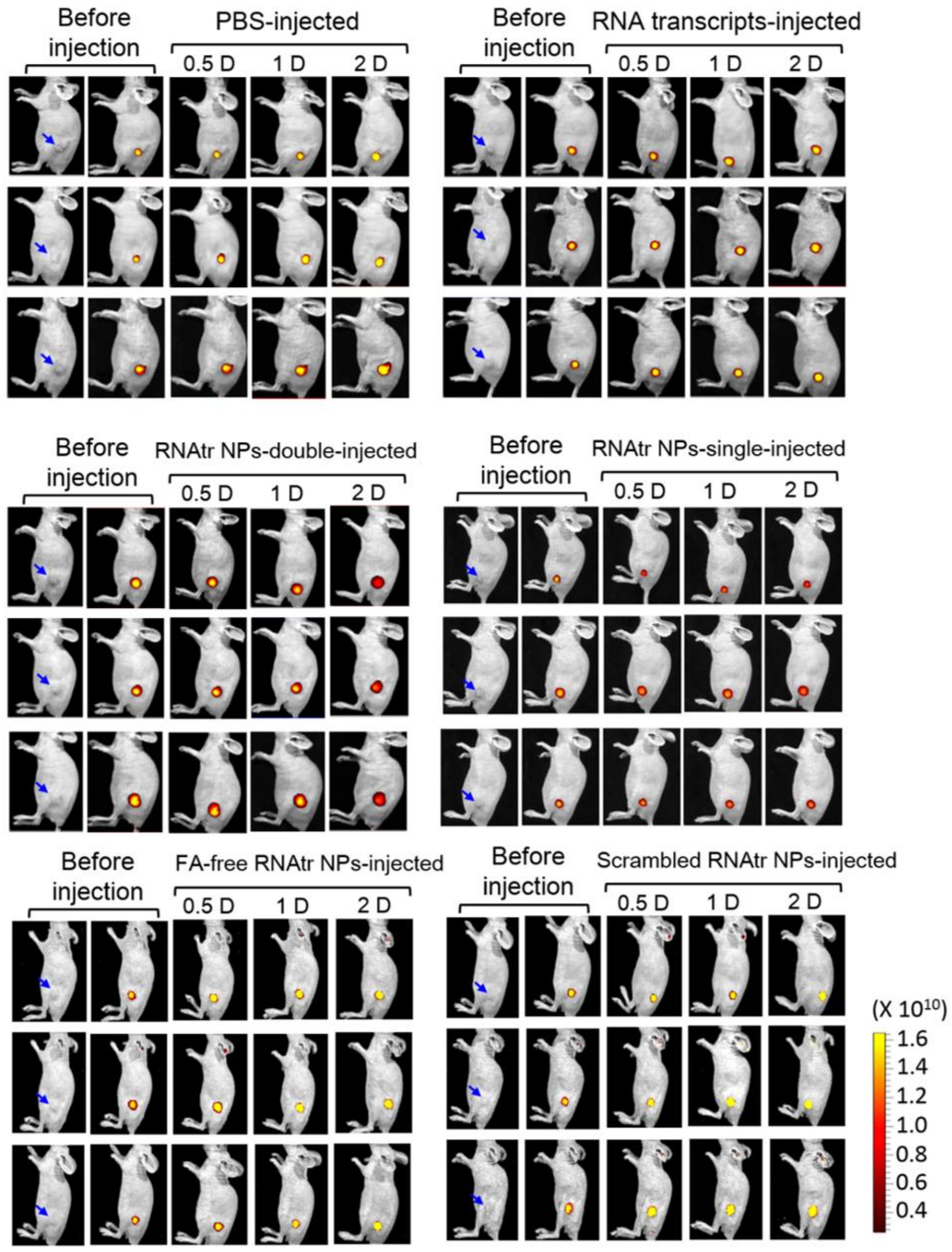

Supplementary Figure 10. ***In vivo* knockdown of RFP expression by RNAtr NPs**. Non-invasive real-time fluorescence images of tumors were monitored after intravenous injection of RNAtr NPs into mice bearing RFP-expressing SKOV3 xenograft tumor. RNAtr NPs-double-injected and RNAtr NPs-single-injected mice indicate that RNAtr NPs were systemically administered at an interval of one day

(days 0, 1; 25 μg per injection) or only once (day 0; 50 μg). Blue arrow = tumor site. As the controls, either PBS or RNA transcripts were systemically administered once (day 0; 50 μg) into mice bearing RFP-expressing SKOV3 xenograft tumor. Also, either FA-free RNAtr NPs or scrambled RNAtr NPs were intravenously injected *via* tail-vein once (day 0; 50 μg). n = 3 mice for each group.

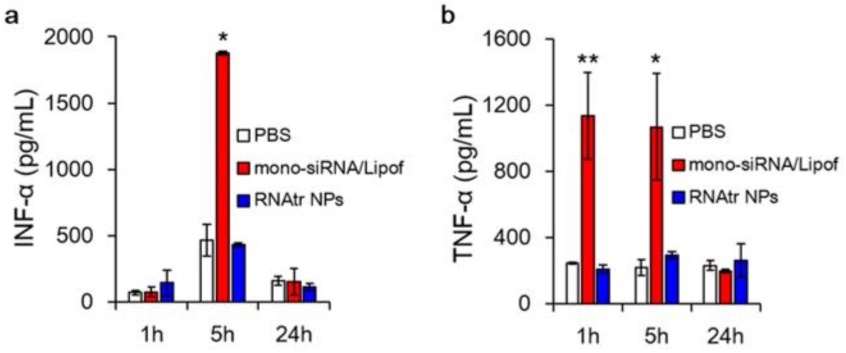

Supplementary Figure 11. Serum immune response for RNAtr NPs from *in vivo* studies. INF-α and TNF-α induction were analyzed 1 h, 5 h and 24 h after intravenous injection with mock (PBS), monomeric siRNA/lipofectamine complexes (equivalent to 200 nM siRNA) or RNAtr NPs (50 μg). The results represent the mean ± s.d. (n = 3). * $p < 0.005$, ** $p < 0.001$ by one-way ANOVA with Tukey`s multiple comparison test, as compared to the PBS control.

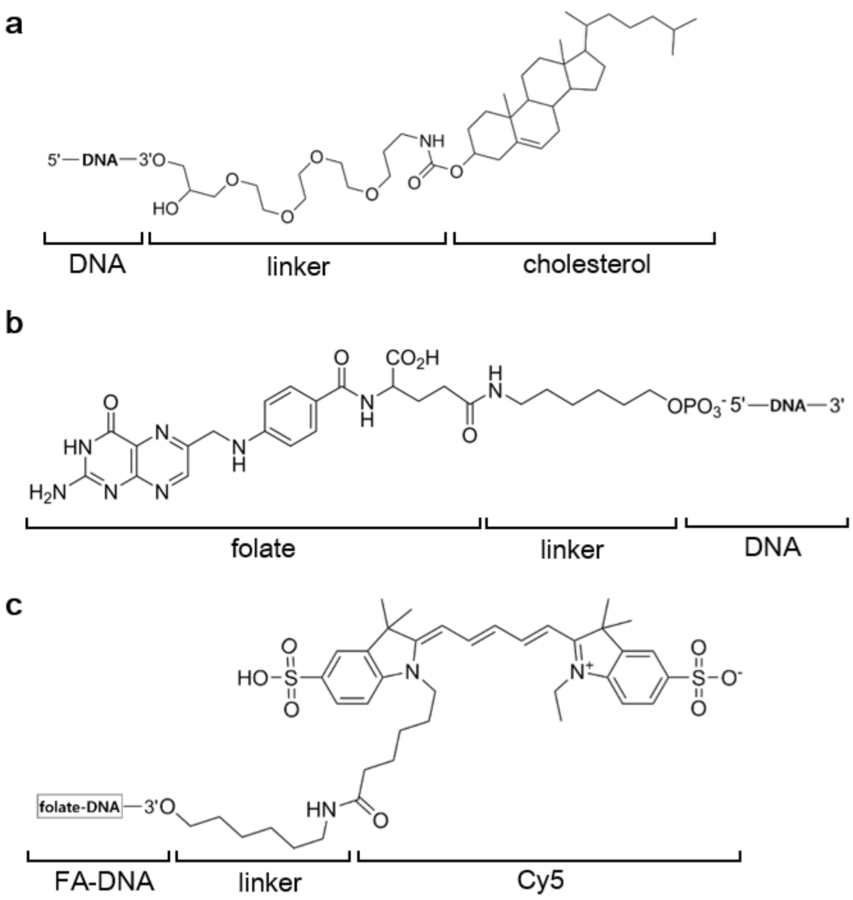

Supplementary Figure 12. Chemical structures of various DNA conjugates. The structures of DNA-Chol (**a**), FA-DNA (**b**) and FA-DNA-Cy5 (**c**), show the sites of conjugation between DNAs and their counterparts as well as the linkers used.

Supplementary Table 1. Oligonucleotide sequences.

Linear DNA template	5'-**ATAGTGAGTCGTATTA**ACGTACCAACAAGAGAGTTCAAGTCCATCTACA ATCTAAAAGTGGTGGGTGTGACCCTAAAATGTAGATGGACTTGAACTCTTTA GAGGCATATCCCT-3'
T7 promoter primer	5'-**TAATACGACTCACTAT**AGGGAT-3'
DNA-Chol conjugate	5'-**ATAGTGAGTCGTATTA**ACGTACCAACAAGA-3'-cholesterol
FA-DNA conjugate	folate-5`-ATCTAAAAGTGGTGGGTGTGACCCTAAAA-3'
anti-RFP siRNA duplex	5'-GAGUUCAAGUCCAUCUACA-3'<br>3'-CUCAAGUUCAGGUAGAUGU-5'
RFP forward primer	5'-GCGTGATGAACTTCGAGGA-3'
RFP reverse primer	5'-GATGAAGCAGCCGTCCTG-3'
β-actin forward primer	5'-AGAGGGAAATCGTGCGTGAC-3'
β-actin reverse primer	5'-CAATAGTGATGACCTGGCCGT-3'
Linear DNA template for scrambled RNAtr NPs	5'-**ATAGTGAGTCGTATTA**ACGTACCAACAAGA<u>GACTTCAAGTGCAACTTCA</u> ATCTAAAAGTGGTGGGTGTGACCCTAAAA<u>TGAAGTTGCACTTGAAGTC</u>TTTA GAGGCATATCCCT-3'

For RNA/DNA base pairing, RNA fragments transcribed from each colored sequences of linear DNA template were rationally designed to be complimentary to the same colored sequences of DNA-Chol or FA-DNA conjugate. Especially, the bold black sequences of linear DNA template are complimentary to partial sequences of T7 promoter primer, and also coincide with the bold yellow sequences in DNA-Chol conjugate. Therefore, RNA fragments transcribed from bold black sequences of DNA template are also involved in hybridizing with DNA-Chol conjugates. The DNA template sequences corresponding to the scrambled sense and scrambled antisense strand are underlined.

Supplementary Table 2. Amount of short RNA generated from RNAtr NPs by Dicer.

	Band intensity (arbitrary unit)	Amount (μg)	
19 bp of anti-RFP siRNA duplex as a standard	12090.6 ± 195.9	1	Reference for measuring the amount of short RNA
Short RNA generated from RNAtr NPs	6315.1 ± 86.3	0.52 ± 0.002	

The band intensity of 1 μg of anti-RFP siRNA duplex, as a standard, was compared with that of short RNA generated from RNAtr NPs by Dicer cleavage *in vitro*, as shown in the Fig. 3d. The amount of short RNA generated from RNAtr NPs was estimated from the known amount of RNA standard. The band intensities corresponding to either standard duplex or short RNA duplex were analyzed by a Gel Doc image analysis system. All experiments were repeated as triplicate.
